# 3D Models of Cellular Spheroids As a Universal Tool for Studying the Cytotoxic Properties of Anticancer Compounds In Vitro

**DOI:** 10.32607/actanaturae.11603

**Published:** 2022

**Authors:** A. S. Sogomonyan, V. O. Shipunova, V. D. Soloviev, V. I. Larionov, P. A. Kotelnikova, S. M. Deyev

**Affiliations:** Shemyakin–Ovchinnikov Institute of Bioorganic Chemistry, Russian Academy of Sciences, Moscow, 117997 Russia; MEPhI (Moscow Engineering Physics Institute), Institute of Engineering Physics for Biomedicine, (PhysBio), Moscow, 115409 Russia; Sirius University of Science and Technology, Sochi, 354340 Russia; Moscow Institute of Physics and Technology (National Research University), Dolgoprudny, Moscow Region, 141701 Russia

**Keywords:** 3D printing, 3D cell culture models, DARPin, TurboFP

## Abstract

The aim of this work is to develop a 3D cell culture model based on cell spheroids for predicting the functional activity of various compounds in vivo. Agarose gel molds were made using 3D printing. The solidified agarose gel is a matrix consisting of nine low-adhesive U-shaped microwells of 2.3 × 3.3 mm for 3D cell spheroid formation and growth. This matrix is placed into a single well of a 12-well plate. The effectiveness of the cell culture method was demonstrated using human ovarian carcinoma SKOVip-kat cells stably expressing the red fluorescent protein Katushka in the cytoplasm and overexpressing the membrane-associated tumor marker HER2. The SKOVip-kat cell spheroids were visualized by fluorescence microscopy. The cell concentration required for the formation of same-shape and same-size spheroids with tight intercellular contacts was optimized. To verify the developed model, the cytotoxicity of the targeted immunotoxin anti-HER2 consisting of the anti-HER2 scaffold DARP 9_29 and a fragment of the Pseudomonas aeroginosa exotoxin, DARP-LoPE, was studied in 2D and 3D SKOVip-kat cell cultures. The existence of a difference in the cytotoxic properties of DARP-LoPE between the 2D and 3D cultures has been demonstrated: the IC_50_ value in the 3D culture is an order of magnitude higher than that in the monolayer culture. The present work describes a universal tool for 3D cultivation of mammalian cells based on reusable agarose gel molds that allows for reproducible formation of multicellular spheroids with tight contacts for molecular and cell biology studies.

## INTRODUCTION

In vitro culturing of mammalian cells remains one of the most valuable tools in molecular and cell biology. In 1885, Wilhelm Roux developed a cell culture method by incubating live chick embryo cells in saline for several days. In 1906, American zoologist Ross Granville Harrison became the first scientist to grow an artificial tissue culture [1]. Cell cultures began to be used as a tool to study the interaction of various substances with living objects as the 19th century was coming to an end [2]. Two-dimensional (2D) cell models, which are currently the main tool employed in in vitro experiments, are widely used in fundamental and applied research; in particular, in developing antitumor therapy methods using various hybrid assemblies [3] and nanoparticles loaded with active substances [4, 5, 6, 7, 8, 9]. Studies in 2D cultures take into account differences from in vivo animal models; however, in order to predict what effect this will have on the body, a large number of cell culture experiments is required. Other disadvantages of monolayer cultures include the lack of a tissue structure and unlimited access of cells to such growth medium components as oxygen, nutrients, and metabolites, while access of a tumor tissue to these substances is, on the contrary, more variable. These limitations have led to the need for an alternative system resembling organs that allows one to perform a large number of routine experiments without laboratory animals. Such systems are spherical clusters of interacting cells: three-dimensional (3D) models [10] such as dense cell aggregations; spheroids grown on the surface of either low-adhesion plastic [11] or agarose [12]; and those obtained using hanging drops [13], alginate capsules [14], and other 3D systems.

Tumor 3D spheroids are closer to in vivo cell models compared to 2D cultures, since the latter do not reflect the architecture of animal organs, which have a specific structure and spatial organization. Spheroids are used to create organelles and organs mimicking the heterogeneity and pathophysiology of oncological processes in a living organism and also test potential drugs [11, 15, 16].

Tumor tissue consists not only of cancer cells but also of stromal cells, such as fibroblasts, vascular endothelial cells, pericytes, adipocytes, lymphatic endothelial cells, and the cells of the immune system. These cells contribute to tumor formation and growth and participate in cancer drug resistance [17]. Spheroids consisting of tumor cells only form cell–cell and cell–extracellular matrix interactions and, thus, create a barrier for the substances to be tested [18]. Therefore, the results of studies of cytotoxic compounds in 3D models differ from those obtained in monolayer cultures. Thus, 3D cultures are most suitable for in vitro studies aimed at predicting and modeling the tumor response to drug exposure. For this reason, introduction of these objects into laboratory practice will save time and costs in identifying new drug candidates, accelerate clinical trials, and reduce the development time to market [18, 19].

This paper presents a simple and universal method for creating 3D spheroids (same-shape and same-size cell clusters) to study the activity of substances in both fundamental and preclinical studies. The 3D printing technique was used to make gel molds from a photopolymer resin. Molds were filled with agarose, which served as the well matrix for cell spheroid formation. Fluorescent microscopy showed the presence of numerous live cells, outnumbering dead ones, during spheroid growth. Comparison of 2D and 3D cell cultures revealed significant differences in the cytotoxicity of the original targeted immunotoxin DARP-LoPE [20]. For instance, the half-maximum inhibitory concentration (IC_50_) value for the immunotoxin in the 3D culture is approximately an order of magnitude higher than that in the 2D culture, which must be taken into account when selecting drug doses for therapeutic injections in vivo.

## EXPERIMENTAL

**Cell culture conditions**

Fluorescent ovarian carcinoma SKOVip-kat cells have been previously obtained to study the effect of antitumor compounds in the intraperitoneal metastasis model in immunodeficient animals [20]. CHO cells were obtained from the collection of the Laboratory of Molecular Immunology of the Institute of Bioorganic Chemistry of the Russian Academy of Sciences. SKOVip-kat and CHO cells were cultured in cell culture flasks (Nunc, Denmark) containing a DMEM medium (Gibco, USA) supplemented with 10% fetal bovine serum (FBS, Capricorn, Germany) in a CO_2_ incubator (BINDER, Germany) at 37°C and 5% CO_2_. The cells were detached from the surface of culture flasks using a Versen solution (PanEco, Russia).

**Formation of fluorescent SKOVip-kat cell spheroids**

Agarose molds were made of a FormLabs Gray Resin 1L photopolymer resin (USA) using a FormLabs Form3 3D printer (USA). Agarose (1%; PanEco) diluted in a colorless Fluorobrite DMEM medium (Gibco) without FBS was used as a mold material for spheroid formation. The spheroids were obtained by adding SKOVip-kat cell suspension to agarose gel wells in a 12-well plate (Nunc) containing the DMEM medium (Gibco) supplemented with 10% FBS (Capricorn) and further culturing of cells for five days in a CO_2_ incubator (BINDER, Germany) at 37°C and 5% CO_2_. The resulting spheroids were stained with fluorescent dyes and visualized using fluorescence microscopes Leica DMI6000B (Leica Microsystems, Germany) and Axiovert 200 (Carl Zeiss, Germany).

**Fluorescence microscopy**

The cells were visualized using fluorescent dyes Hoechst 33342 (PanEco), propidium iodide, and acridine orange (Sigma-Aldrich, USA).

Labeled SKOVip-kat spheroids were visualized using the inverted fluorescence microscopes Leica DMI6000B and Axiovert 200. The Katushka (TurboFP635) protein fluorescence was excited with the HBO 100W mercury lamp of an Axiovert 200 fluorescence microscope with excitation and emission wavelengths of 565/30 and 620/60 nm, respectively; the excitation and emission wavelengths for fluorescent dyes were 365/12 and 397/LP nm for Hoechst 33342 and 565/30 and 620/60 nm for propidium iodide, respectively. The Katushka protein fluorescence was also excited using the metal halide lamp of a Leica DMI6000B fluorescence microscope with excitation and emission wavelengths of 545/30 and 610/75 nm, respectively; the excitation and emission wavelengths for fluorescent dyes were 405/10 and 460/40 nm for Hoechst 33342, 545/30 and 610/75 nm for propidium iodide, and 470/40 and 525/50 nm for acridine orange, respectively. Plastic 96-well plates (Nunc) were used to visualize the 2D SKOVip-kat and CHO cell cultures. The cells were incubated in 100 µL of a colorless DMEM medium (Gibco) with FBS (Capricorn) for 12 h at 37°C and 5% CO_2_. Then, either the monoclonal antibody trastuzumab or DARP-LoPE immunotoxin conjugated to the fluorescent dye fluorescein 5(6)-isothiocyanate (FITC) was added to a final concentration of 2 μg/mL [7] in a volume of 100 μL. The cells were washed to remove unbound proteins and resuspended in a 1% bovine serum albumin in phosphate buffer. A Leica DMI6000B fluorescence microscope was used for visualization.

**Cell viability assay**

The cytotoxicity of the SKOVip-kat [20] and CHO cells incubated with DARP-LoPE immunotoxin [21] was analyzed using the colorimetric MTT assay (MTT is a yellow tetrazolium dye that is reduced to purple formazan by live cells) [22].

The assay was performed in a 96-well plate (Nunc). The SKOVip-kat and CHO cells (3.5 × 10^3^ cells per well) were incubated in 100 µL of a phenol-red free DMEM medium (Gibco (Thermo Scientific), USA) supplemented with 10% FBS (Capricorn) for 12 h at 37°C and 5% CO_2_. Then, 100 µl of DARP-LoPE immunotoxin was added and the cells were incubated for 72 h. After this, the medium underwent shaking and 100 µl of 0.5 g/L MTT were added. The MTT solution underwent shaking after 1 h, and 100 µL of DMSO (Panreac-AppliChem, USA) was added to the wells to dissolve formazan. The optical density was measured using an Infinite M1000 Pro microplate reader (Tecan, Austria) at a wavelength of 570 nm and a reference wavelength of 630 nm. The IC_50_ values of DARP-LoPE in SKOVip-kat and CHO cells were determined using the GraphPad Prism 8.0.1 software.

## RESULTS AND DISCUSSION

The aim of the current work is to produce reproducible 3D spheroids in vitro that mimic the characteristics of tumor tissues to test various active substances, including drugs. We used human ovarian carcinoma SKOVip-kat cells overexpressing the HER2 receptor, a diagnostic and therapeutic marker of some cancers, on its surface. This cell line has been previously obtained by stably transfecting SKOV3-1ip cells with the gene of the red fluorescent protein Katushka [23]. The Katushka fluorescence excitation and emission wavelengths are in the near infrared region (588 and 635 nm, respectively) [24]; this region falls in the tissue transparency window, which makes it possible to visualize these cells both in vitro and in vivo with equal efficiency.

**Formation of 3D spheroids using agarose molds**

Agarose, which is a natural biodegradable, non-adhesive, and non-toxic polysaccharide derived from seaweed, was used as the matrix for the 3D spheroids [25]. Agarose has the characteristic necessary for creating three-dimensional cell culture models: high porosity (average pore size, 100–300 nm), which allows for the renewal of nutrient media for 3D cell growth [25] and provides access to gases and small molecules [26]. Since agarose is an optically transparent material, it is suitable for the microscopic visualization of spheroids. Agarose gel solidifies in molds at room temperature, which makes it possible to perform experiments under sterile conditions without significant difficulties, while the accessibility of the resulting gel wells to a pipette tip makes it possible to introduce cells and conduct other mold manipulations.

The resulting agarose molds have nine identical wells, 2.3 mm in diameter and 3.3 mm in height, in which spheroids with the same size and shape are formed. The designed mold is an open system that allows one to analyze spheroid formation and test various compounds using light and fluorescence microscopy.

**Fig. 1:**
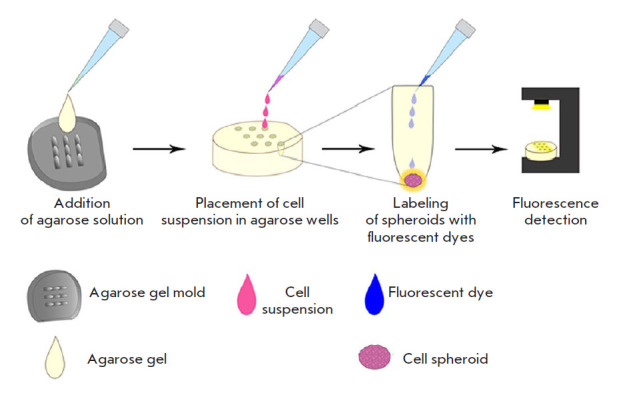
Design of 3D SKOVip-kat cell spheroids. An agarose solution was added to the molds for solidification at room temperature. A suspension of SKOVip-kat cells was added to the gel-containing wells. After the spheroids had formed, the cells were labeled with fluorescent dyes and analyzed by fluorescence microscopy

**Fig. 2:**
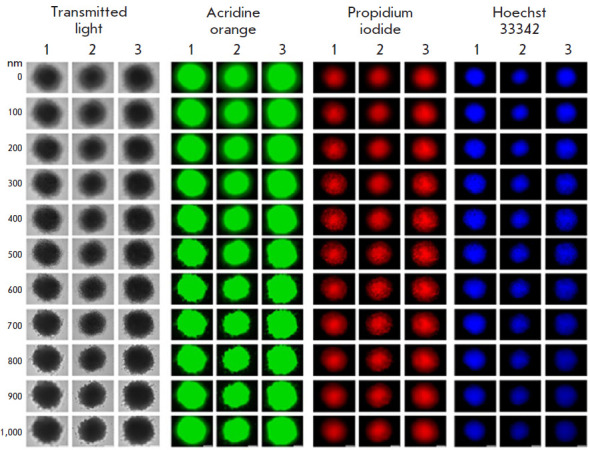
Imaging of SKOVip-kat spheroids. Imaging of three representative spheroids stained with the fluorescent dyes acridine orange, Hoechst 33342, and propidium iodide with a Z-axis step of 200 nm. The excitation and emission wavelengths for fluorescence detection were as follows: 470/40 and 525/50 nm for acridine orange, 545/30 and 610/75 nm for propidium iodide, and 405/10 and 460/40 nm for Hoechst 33342, respectively. Scale: 250 μm

Figure 1 presents the design of SKOVip-kat cell spheroids. Molds for the agarose gel were printed on a FormLabs Form3 3D printer (USA) using the FormLabs Gray Resin (USA). The agarose volume in the mold is 1,200 µl; the volume of a single agarose well is 10 µl. The agarose surface is non-adhesive to cells, which allows for spheroid self-formation. Spheroids formed in five days, which was confirmed visually by the presence of intercellular contacts [26] (Fig. 2). Figure 2 shows the viability of the cells inside the spheroids assessed using a Leica DMI6000B fluorescence microscope. Three representative spheroids stained with fluorescent dyes were visualized along the Z axis with a 200-nm step. The fluorescent dye acridine orange stains nucleic acids in living cells; propidium iodide stains nucleic acids in dead cells, since the membranes of living cells are impermeable to the dye [27]; Hoechst 33342 stains nucleic acids in nuclei [28] by passing through the membranes of living cells [29]. Staining with acridine orange and Hoechst 33342 showed that there are more live cells than dead cells stained with propidium iodide both inside and outside the spheroid. Thus, the 3D cell cultures obtained by us are most suitable for testing drugs, since the cells in a spheroid create intercellular contacts and create an approximate model of cancer tissues; i.e., they represent a more adequate in vitro system than 2D cultures.

**Evaluation of HER2 receptor expression on the SKOVip**-**kat cell surface **

**Fig. 3:**
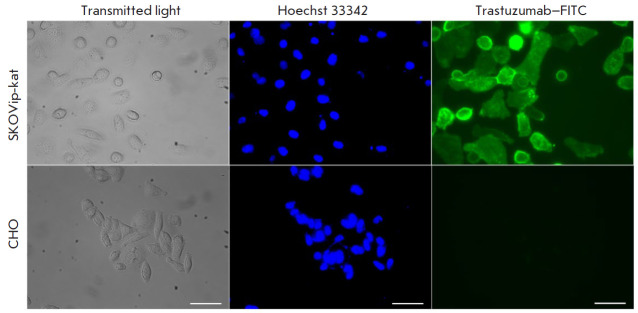
Imaging of the HER2 receptor expression in SKOVip-kat (HER2-positive) and CHO (HER2-negative) cells using the monoclonal antibody trastuzumab conjugated to the fluorescent dye FITC. Expression of HER2 on the SKOVip-kat cell surface was confirmed by intense staining of the cell membrane with the anti-HER2 antibody. Cell nuclei were stained with Hoechst 33342. The excitation and emission wavelengths for fluorescence detection were as follows: 405/10 and 460/40 nm Hoechst 33342 and 470/40 and 525/50 nm for FITC, respectively. Scale: 50 μm

HER2 (human epidermal growth factor receptor 2) is a well-known membrane-associated tumor marker [30, 31, 32]. Expression of this receptor is often high in mammary, ovarian, endometrial, gastric, and esophageal cancers and low in normal cells [33]. For example, this tumor marker is found in 30% of breast cancers [34]; for this reason, HER2 is considered an important target in tumor diagnosis and therapy. HER2 expression on the surface of SKOVip-kat cells was evaluated using the monoclonal antibody trastuzumab conjugated to FITC. Chinese hamster ovary CHO cells lacking HER2 on their cell surface were used as a negative control (Fig. 3). Both cell cultures were incubated with a trastuzumab–FITC conjugate and then visualized on a Leica DMI6000B fluorescence microscope. The data presented in Fig. 3 confirm the presence of HER2 on the SKOVip-kat cell surface.

**DARP-LoPE immunotoxin cytotoxicity in the 2D culture**

In order to validate the developed 3D model as a tool for studying the antitumor efficacy of the compounds, we evaluated the cytotoxicity of the targeted antitumor compound, DARP-LoPE immunotoxin.

Immunotoxins are targeted proteins fused to the toxin isolated from either bacteria or poisonous plants [35, 36]; they are considered one of the most promising targeted molecules in oncotherapy. The immunotoxin DARP-LoPE has previously been genetically engineered using the non-immunoglobulin designed ankyrin repeat protein DARP 9_29 that binds to the HER2 receptor [37, 38], and the low-immunogenic variant of the exotoxin A region (LoPE) isolated from the Gram-negative bacterium Pseudomonas aeruginosa [21]. This immunotoxin binds specifically to HER2 and induces tumor cell death in vitro [21]. Moreover, DARP-LoPE effectively inhibits the growth of HER2- positive human ovarian carcinoma xenografts, which confirms the effectiveness of DARPin-based targeted drugs [5, 20, 21, 39].

**Fig. 4:**
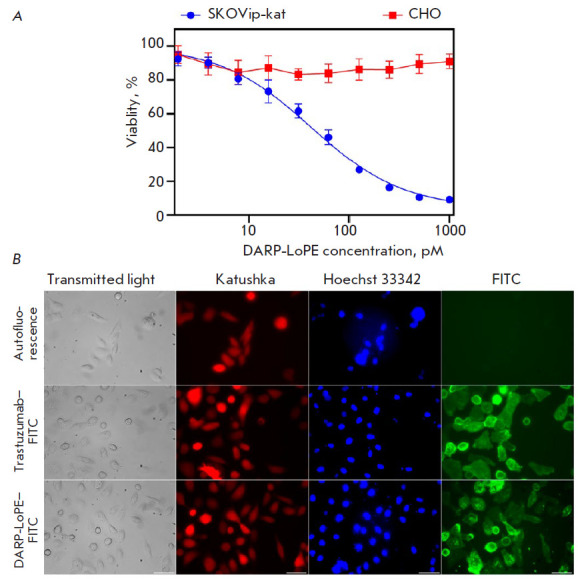
Interaction of the targeted immunotoxin DARP-LoPE with SKOVip-kat cells. (*A*) – evaluation of DARP-LoPE cytotoxicity in SKOVip-Kat and CHO cells using the MTT assay. Cell viablity in the absence of DARP-LoPE immunotoxin was considered as 100%. (*B*) – visualization of live cells using the Katushka protein (TurboFP635) and Hoechst 33342 dye; visualization of HER2 receptor expression in SKOVip-kat cells incubated with the monoclonal antibody trastuzumab–FITC and immunotoxin DARP-LoPE–FITC. The excitation and emission wavelengths were as follows: 545/30 and 610/75 nm for Katushka protein, 405/10 and 460/40 nm for Hoechst 33342, and 470/40 and 525/50 nm for FITC, respectively. Scale: 50 μm

Figure 4 shows DARP-LoPE cytotoxicity analysis results and fluorescence microscopy data confirming the specificity of immunotoxin binding to SKOVip-kat cells. Cytotoxicity was evaluated using the MTT assay; the data was processed using the OriginPro 2015 software. The obtained results indicate the targeted cytotoxicity of DARP-LoPE in SKOVip-kat cells and the absence of DARP-LoPE cytotoxicity in CHO. The IC_50_ value for DARP-LoPE in SKOVip-kat cells was 41.9 pM (Fig. 4A).

Tumor cells were visualized by labeling HER2 on the surface of SKOVip-kat cells with the monoclonal antibody trastuzumab and FITC-conjugated DARP-LoPE. It was shown that both immunotoxin and trastuzumab effectively interact with HER2 on the tumor cell surface (Fig. 4B).

**DARP-LoPE immunotoxin cytotoxicity in SKOVip-kat spheroids**

**Fig. 5:**
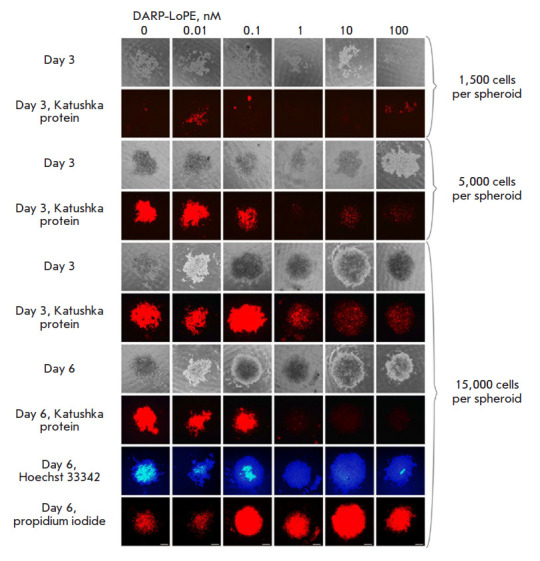
Imaging of SKOVip-kat spheroids and analysis of DARP-LoPE immunotoxin cytotoxicity in the 3D culture. The cells were incubated with various concentrations of immunotoxin; cell viability was analyzed for six days. The cytotoxicity of DARP-LoPE immunotoxin in the 3D cell culture containing spheroids comprised of a different number of SKOVip-kat cells was analyzed. The optimal number of cells for creating a 3D culture was shown to be 15,000 cells per spheroid. The viability of SKOVip-kat cells was assessed based on the fluorescence of the Katushka protein using real-time fluorescence microscopy on days 3 and 6. On day 6, the spheroids were incubated with dyes: live and dead cells were visualized using Hoechst 33342 and propidium iodide, respectively. The Excitation and emission wavelengths were as follows: 565/30 and 620/60 nm for Katushka, 365/12 and 397/LP nm for Hoechst 33342, and 565/30 and 620/60 nm for propidium iodide, respectively. Scale: 250 μm

In order to select the optimal number of cells in the spheroid wells, the concentration range from 1,500 to 15,000 cells per well was tested. Optimal concentrations were determined on day 3 of cell incubation in the agarose wells by transmitted light microscopy and fluorescence visualization of the Katushka protein in SKOVip-kat. Reproducibility of our results and formation of cell contacts (the absence of cell fragmentation) [26] were observed in wells containing 15,000 cells per spheroid (Fig. 5).

Along with selection of the cell concentrations, DARP-LoPE cytotoxicity was studied by incubating the spheroids with various concentrations of DARP-LoPE. After incubation with the protein and staining with Hoechst 33342 and propidium iodide, the samples were analyzed by fluorescence microscopy (Fig. 5). Visually determined IC_50_ of DARP-LoPE in the 3D culture was 0.3 nM, which is about eight times greater than that in the 2D culture (41.9 pM). Since the structural organization of 3D cell models is closer to animal models in vivo than that of 2D models, the visualization and cytotoxicity results in the 3D culture should presumably be similar to those obtained in animal objects in vivo.

## CONCLUSION

The transition from 2D to 3D models is necessary due to the insufficient information value of 2D systems when studying various effects and testing drugs for the diagnosis and treatment of various diseases. The creation of 3D spheroids imitating solid tumors and their introduction in research practice can also be rationalized on ethical grounds: the results obtained by using these systems are closer to in vivo results [40]. Thus, the use of these models may reduce the number of animal experiments required for drug screening [41].

Three-dimensional cell spheroids form a specific microenvironment with characteristics different from those of 2D structures: pH value, presence and concentration of autocrine factors, as well as oxygen and CO_2_ concentrations; cells in this microenvironment have their own morphology, ability to differentiate, proliferate, and respond to various stimuli, thereby imitating the in vivo behavior. These properties of cells in a spheroid are important in order to study the effect of various drugs, since the artificial microenvironment limits penetration of the latter; therefore, a higher substance concentration is required to achieve the desired effect [18].

In our work, we present a method for creating cancer cell spheroids based on 3D printing of photopolymer resin molds and their filling with agarose. It is a simple and reproducible method for drug testing; it allows one to obtain cytotoxicity analysis results that are close to those obtained in vivo. Today, 3D printing is becoming an affordable means for obtaining molds with the desired characteristics; it is widely used in various fields, such as regenerative medicine [42], engineering [43], architecture [44], and manufacturing [45]. To date, 3D printers and materials for creating the desired objects have become more affordable [46], which makes it possible to use the technique in many laboratories. The use of agarose as the matrix for spheroid formation makes this method as effective as possible for routine experiments. Since agarose is low adhesive to cells, interactions in a spheroid occur only between cells, which promotes cell growth in all directions instead of just one. In addition, since agarose is a transparent polymer, it can be used in various studies: in particular, in photodynamic therapy. Furthermore, the developed spheroid model is an open system that allows one to perform such cell manipulations as medium change and washoff of various components, external exposure to electromagnetic radiation, introduction of other cell types (endothelium cells and fibroblasts), and placement of biopsy specimens into a separate well.

Using the developed method, we obtained reproducible same-shape and same-size 3D spheroids from fluorescent SKOVip-kat cells. Significant differences were revealed in the effect of the targeted immunotoxin between 2D and 3D models using the colorimetric toxicity assay and fluorescence microscopy. Thus, we have developed a simple and effective method for obtaining representative 3D spheroid models for molecular biological and cellular studies [47, 48].

## Abbreviations

MTT: 3-(4,5-dimethylthiazol-2-yl)-2,5-diphenyltetrazolium bromide
FITC: fluorescein isothiocyanate
DARPin: designed ankyrin repeat protein
LoPE: low immunogenic exotoxin A fragment of the gram-negative bacteria Pseudomonas aeruginosa
HER2: human epidermal growth factor receptor 2
